# Building with graphene oxide: effect of graphite nature and oxidation methods on the graphene assembly†

**DOI:** 10.1039/d0ra10207e

**Published:** 2021-01-18

**Authors:** Ji Hoon Kim, Gyu Hyeon Shim, Thi To Nguyen Vo, Boyeon Kweon, Koung Moon Kim, Ho Seon Ahn

**Affiliations:** Department of Mechanical Engineering, Incheon National University Incheon 22012 Republic of Korea hsahn@inu.ac.kr; Research Institute of Basic Sciences, Incheon National University Incheon 22012 Republic of Korea; AHN Materials Inc Incheon 22012 Republic of Korea

## Abstract

During nearly 2 centuries of history in graphene researches, numerous researches were reported to synthesize graphene oxide (GO) and build a proper graphene assembly. However, tons of research prevail without verifying the reproducibility of GO that can be sensitively attributed by the graphite nature, and chemical processes. Here, the structure and chemistry of GO products were analyzed by considering parent graphite sources, and three different oxidation methods based on Hummer's method and the addition of H_3_PO_4_. The oxidation level of GO was characterized by monitoring the C/O and sp^2^ carbon ratio from X-ray photoelectroscopy (XPS) spectra. It was observed that the oxidant intercalation behavior was dependent on the morphological differences of graphite; synthetic and natural flake graphite were compared based on their origins in shape and size from different suppliers. Thermal reduction and exfoliation were applied to GO powders to prepare thermally expanded graphene oxide (TEGO) as a graphene assembly. Gas releases from the reduction of oxygen functional groups split layered GO structure and build a porous structure that varied specific surface area regarding oxidation degrees of GO.

## Abstract 2

In this study, the article investigated the effect of starting graphite sources, and oxidation methods on graphene oxide (GO) synthesis and the porous structure of building assembly into thermally expanded graphene oxide (TEGO).

## Graphical abstract

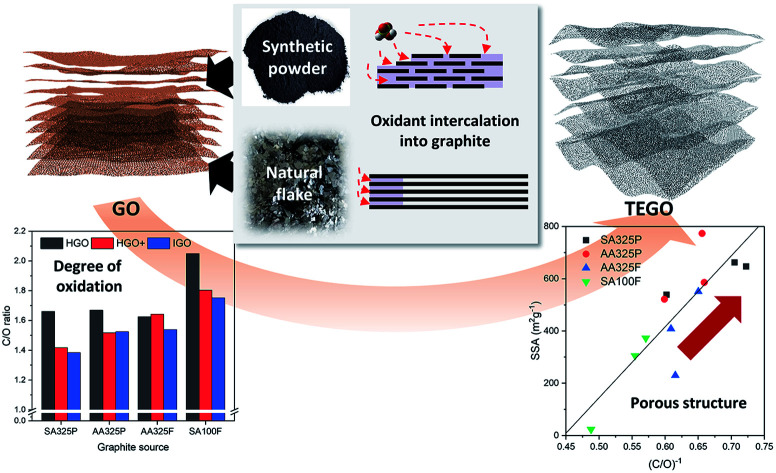

## 1. Introduction

Since graphene was discovered, its novel and unique properties attracted tremendous global interest in many applications such as energy storage,^1^ catalysts for environmental protection,^2,3^ and bioelectrochemical^4^ applications. Based on a top-down approach, graphene can be prepared by chemical and mechanical exfoliation of graphite sources. GO synthesis is a popular wet chemical method due to its potential scalability, high yield and excellent dispersibility in various solvents.^5^ Over the years, GO has been extensively studied for understanding its fundamental chemical structures^6–8^ and formation mechanisms with various synthetic methods.

In chemical exfoliation, GO synthesis normally begins by intercalating a strong oxidant and concentrated acidic solution to oxidize the graphite raw material. So, the graphite nature should be carefully considered as one of the factors which dominantly affects the state of the final GO product. Depending on a source, graphite can be obtained naturally or synthetically. Natural graphite is classified by vein, flake, and amorphous graphite, which has macrocrystalline and is found in a great number of mineral origins.^9^ Among them, the flake graphite has been used as the main source for GO synthesis research and development due to good crystallinity.^10^ Synthetic graphite is a man-made substance manufactured by the high-temperature processing of amorphous carbon materials. Synthetic graphite has a somewhat less crystalline structure than natural graphite.^11^

The first synthesis of GO reported over 160 years ago when B. Brodie^12^ treated graphite with strong oxidizers KClO in fuming nitric acid, successfully producing the purest and most stable GO. The use of oxidant KClO was replaced with less dangerous and more convenient oxidizing reagents by Staudenmaier and Hofmann.^13,14^ Recently, Hummers' permanganate oxidation^15^ and its modified method^16^ have become the most representative method due to the safety and higher degree of oxidation, *e.g.* normally 2.25 of C/O ratio. However, the traces of sulfur (up to 6%),^17^ nitrogen impurities^18^ and defects due to diol cleavages^19^ were regarded as remaining problems of Hummers' method; many studies have been reported to identify the GO formation mechanism.^20–22^ To resolve these chronic problems in GO formation, Tour's group applied the use of H_3_PO_4_ as the second acid instead of NaNO_3_ called ‘improved method’.^23^ It was reported that the regular structure could be attained because of the formation of five-membered cyclic phosphate groups between the H_3_PO_4_ and two vicinal diols (C–OH) on the graphene basal plane.

**Fig. 1:**
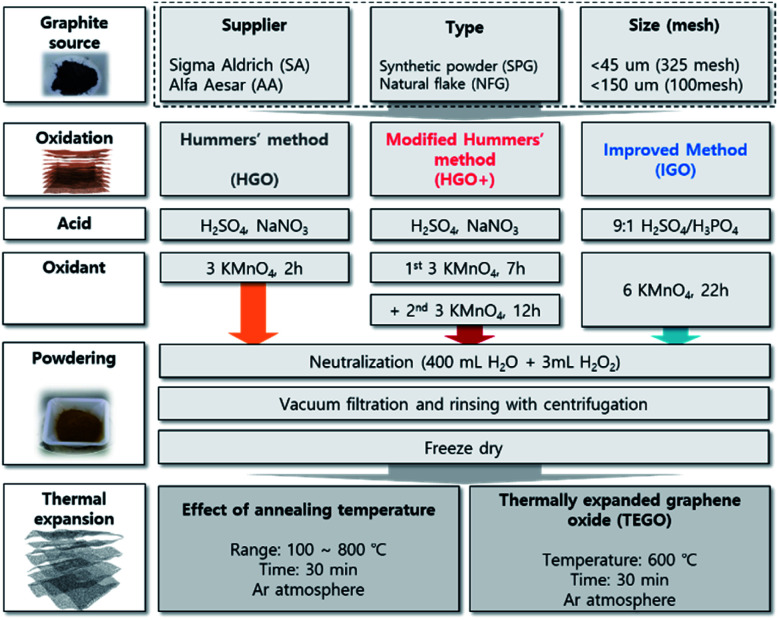
Schematic illustration of the experimental procedures and their features.

Despite efforts of many reviews and excellent studies on GO, its formation mechanism is still unclear, and there are many factors to be considered in whole synthetic stages including oxidation, neutralization, filtration, rinsing, and drying. From an engineering perspective, these uncertainties in synthesis present serious difficulties in ensuring the reliability and reproducibility of the material. In this study, the article is dealing with the effect of starting graphite sources, and three different oxidation methods on the GO preparation with carefully conducted treatment of oxidized mixture after reaction. The prominent oxidation methods including Hummers' (HGO) and modified method (HGO+), and improved method (IGO) were applied to graphite sources that purchased from a different supplier with different originality: synthetic powder graphite (SPG), and natural flake graphite (NFG) that have difference in crystalline structure, in-plane order in graphene, and purity. Structural differences in GO powders were compared by microscopic observation, concerning the intercalation of oxidant into the graphitic interlayers. The oxidation level was monitored by the atomic C/O ratio, and the chemical composition of GO powders. The structural uniformity was checked *via* Raman spectra and the defect ratio in the graphene basal plane. The GO powders were converted to TEGO by thermal expansion method (Fig. 1), and the pore structures were compared by microscopic structural analysis, and quantitative analysis *via* N_2_ isotherm at 77 K.

## 2. Experimental

### 2.1. Synthesis of graphene oxide

#### 2.1.1. Hummers' method (HGO)

Four graphite sources from two different suppliers (Sigma-Aldrich; SA and Alfa Aesar; AA) were used with respect to their size (100 and 325 mesh) and shapes (synthetic powder and flake) (Table S1†). Graphite powder (2 g) and NaNO_3_ (1 g, ≥99.0%; Sigma-Aldrich) were added to concentrated H_2_SO_4_ (69 mL, 95%; Daejung) in a triple neck flask with 500 mL of capacity and we stirred them under 800 rpm of rotating octagonal stir bar while maintaining the temperature below 10 °C using an ice bath. Oxidizing reagent KMnO_4_ (6 g, ≥99.0%; Daejung) was dropped into a reaction flask for 10 minutes and reacted carefully for 2 hours so that the temperature of the mixture did not exceed 20 °C. The mixture was stirred for 2 hours as the temperature was maintained at 35 °C. We then added the solution, which was mixed with deionized water (400 mL) and H_2_O_2_ (3 mL, 30%; Daejung), using a biuret with 1200 rpm of rotation speed and maintaining the temperature of the mixture below 10 °C. After sifting with test sieve (300 μm, ASTM E11), the GO cake was obtained by vacuum filtering the sludge from the previous process. To remove contaminations, multiple steps of rinsing processes are carried out, including centrifugation by 4000 rpm of rotating speed. The precipitated as-prepared GO was collected and filtered again to get the GO cake, and dehydrated by freezing dry for 48 h. Finally, GO powder was obtained by grinding GO cake in a bowl.

#### 2.1.2. Modified Hummers' method (HGO+)

The modified Hummers' method (HGO+) was conducted by the increased amounts of reagents and reaction times that divided by 2 steps. Increased amount of graphite powder (3 g) and NaNO_3_ (1.5 g) were dropped to concentrated H_2_SO_4_ (69 mL, 95%) with the same reaction environment used in HGO. 9 g of KMnO_4_ was firstly dropped into the reaction flaks for 10 minutes and reacted for 7 hours under 35 °C. As the second step, 9 g of KMnO_4_ was additionally dropped into the flask and the mixture was reacted for 12 hours at 35 °C. Then, the same post-procedures corresponding to HGO was performed to obtain HGO+ powders, *i.e.* neutralization, vacuum filtration, rinsing, centrifuge, and freeze dry.

#### 2.1.3. Improved method (IGO)

3 g of the graphite source was mixed with a 9 : 1 concentrated H_2_SO_4_/H_3_PO_4_ (360 : 40 mL). KMnO_4_ oxidant was dropped into the mixture under vigorous stirring with 800 rpm of rotation speed for 10 minutes and the reaction continued at 50 °C for 22 h. DI water (400 mL) and H_2_O_2_ (3 mL, 30%) were added to neutralize the sludge as same in the Hummer's method with 1200 rpm of rotation speed in the ice bath at 0 °C. The rinsing, filtration, dehydration, and grinding procedures were similarly performed the same as Hummers' method.

### 2.2. Thermal expansion of GO

The GO powders were thermally treated in a quartz tubular furnace with argon gas atmospheric conditions (0.5 L min^−1^) at the annealing temperature from 100 to 800 °C (5 °C min^−1^ of increment) for 30 min. Black colored TEGO powder was obtained by gas release during thermal reduction.

### 2.3. Characterization

The microstructures of the samples were observed by scanning electron microscopy (SEM: JSM-7800F, 15 kV; JEOL), and transmitted electron microscopy (TEM: F200X, accelerated at 200 kV; Talos). The crystalline structure of GO was characterized by X-ray diffraction (XRD: SmartLab; Rigaku, 9 kW, Cu target with 1.5412 Å of wavelength) pattern, and RAMAN spectroscopy (Alpha-300, excited by a 532 nm Nd:YAG laser, 700 nm of spot size; Witec). The oxygen functional groups of GO and TEGO were analyzed by XPS spectra of the palletized samples (PHI 5000 VersaProbe II; Ulvac). The C1 XPS spectra were deconvoluted by XPSpeak 4.1. The pore structure of TEGO was quantitatively characterized by Brunauer–Emmet–Teller (BET) analysis (Micromeritics, ASAP2020) on N_2_ adsorption isotherm at 77 K.

## 3. Results and discussion

### 3.1. Morphology of GO powders according to graphite and oxidations

GO powders were prepared by HGO, HGO+, and IGO methods from multiple graphite sources. After the oxidation was completed, the gray graphite turned to dark brown GO powder (Fig. S1†). Compared to the dark brown HGO, the appearance of GO powders turned to be brighter in the order of HGO+, and IGO.

**Fig. 2:**
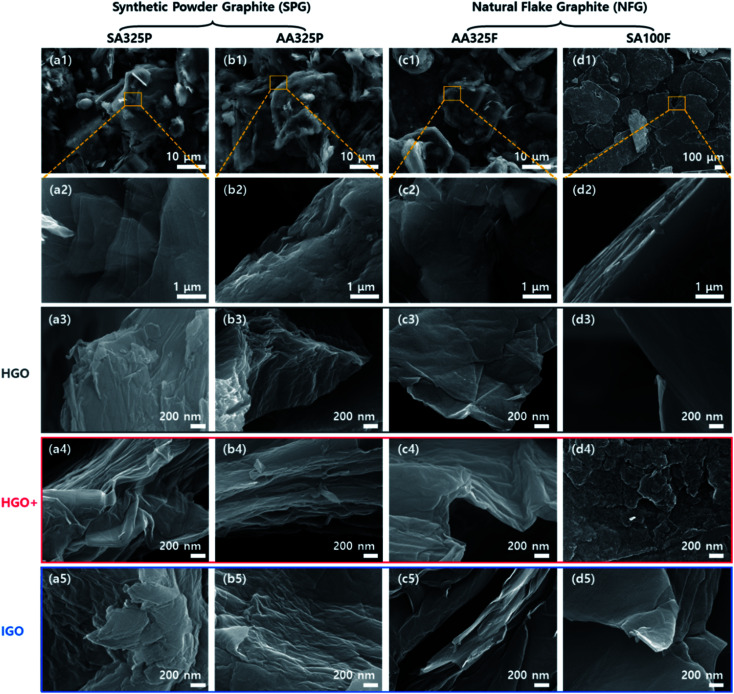
SEM image classified according to graphite source; (a–d) SA325P, AA325P, AA325F, and SA100F, respectively. (1 and 2) Graphite with magnification of ×1000 and ×20 000. (3–5) High magnification SEM image (×50 000) of GO produced by different oxidation methods: HGO, HGO+, and IGO.

Structures of graphite sources and GO powders were characterized by SEM images (Fig. 2), regarding oxidation methods and graphite sources. An elongated acicular and anisotropic grains are observed in the SPGs (SA325P and AA325P) due to the graphitization process from the supplier (Fig. 2a(1) and b(1)). In the case of the NFGs (AA325F and SA100F), the microstructure consists of small layers without preferred orientation. The average sizes of the primary particles of SA325P, AA325P, and AA325F are lower than 45 μm. The sizes of SA100F particles are up to 600 μm, and only the edge of flake is exposed, showing layered structure (Fig. 2d(2)).

**Fig. 3:**
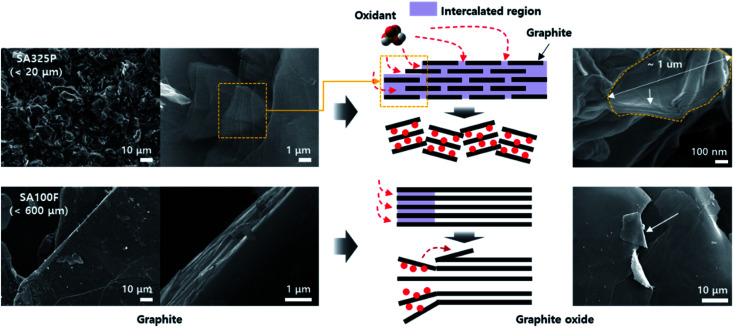
Source dependent oxidant intercalation behavior into the graphitic interlayers of SA325P and SA100F. The oxidant fully intercalated in the SPG-based smaller graphite source, rather partial intercalation in the larger NFG-based source.

The microstructures of GO powders were analyzed by low and high magnification SEM image as shown in Fig. 2 and S2.† Maintaining the acicular grain shape, the GO powders converted from SA325P, and AA325P tend to become more wrinkled and partially exfoliated structure regardless of the oxidation method as shown in Fig. 2a(3–5) and b(3–5). The edge of GO powders converted from AA325F, and SA100F was less wrinkled (Fig. 2c(3–5) and d(3–5)), and the macrostructure was delaminated due to the shear force during grinding. Fig. 3 displays the conversion of graphite to GO according to the initial graphite size. Because of the smaller size of SA325P, the primary particle exposes more graphitic boundary that can lead higher oxidant intercalation into the internal graphitic interlayers. In the case of SA100F, the edge of flake was folded that might be evidence of partial oxidation locally concentrated at the periphery of primary particle. Hence, the GO cakes from SPG was split into smaller particle which was favorable to oxidant intercalation (Fig. S2a(3–5) and b(3–5)†).

### 3.2. Chemical and crystalline structure of GO powder

**Fig. 4:**
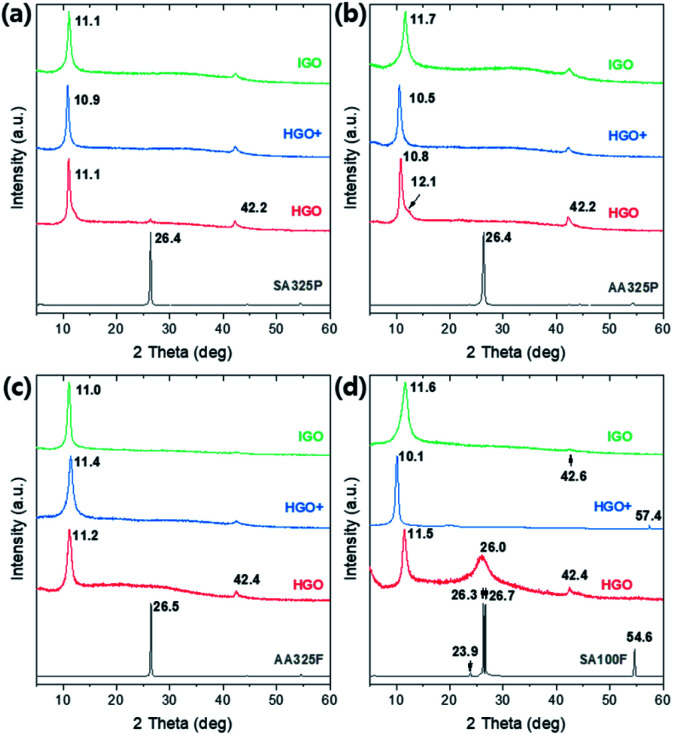
XRD patterns of graphite sources, and the GO powders converted from (a) SA325P, (b) AA325P, (c) AA325F, and (d) SA100F, respectively.

The crystalline structure of graphite and GOs were characterized by the XRD patterns (Fig. 4). A sharp peak is observed on ∼26.4 degrees in all graphite sources, which indicates (002) lattice with spacing *d*_002_ ∼ 3.375 Å. Double peaks are found in the only SA100F due to broad and large distribution of particle sizes. Set of small peaks near 42–45 degrees correspond (101) and (100) lattices of in-plane graphitic sp^2^ crystals (*d*_101_ ∼ 2.14 Å and *d*_100_ ∼ 2.04 Å) which are equally retained in GO samples. After the oxidations, a sharp peak is shifted to 10–12 degrees, corresponding (001) lattice which directly interprets that the GOs are clearly oxidized and the crystalline structures are expanded due to intercalation of oxygen functional groups. The spacings of the whole samples are calculated by Bragg's law and summarized in Table S2.† However, we could not find a specific relationship on crystalline spacings between oxidation methods. The XRD spectra of SA325P-HGO and SA100F-HGO show peak at ∼26.0 degrees due to partially not oxidized traces of graphite (Fig. 4d). In the case of SA100F-HGO, the peak at 26.0 degrees is broader, indicating that the larger size of SA100F is hard to be oxidized because the oxidant did not fully intercalate graphitic layers with lower oxidant uses.

**Fig. 5:**
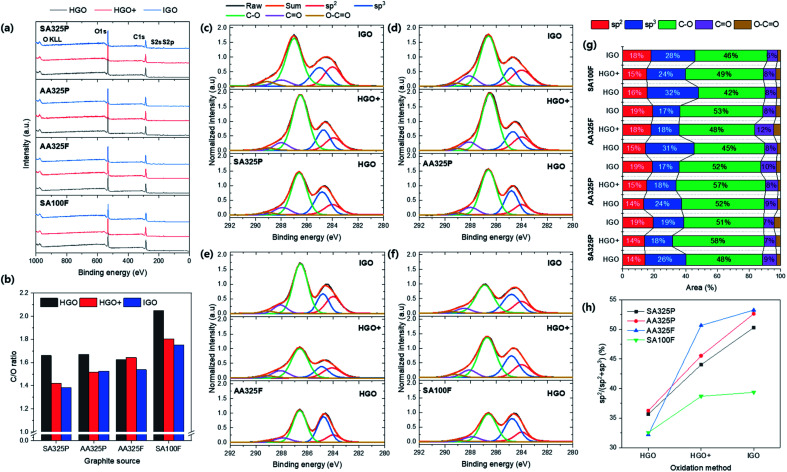
XPS spectra of GO powders. (a) Survey spectra. (b) The C/O ratio of GO powders. (c–f) The C 1s XPS spectra of GO regarding graphite sources: SA325P, AA325P, AA325F, and SA100F, respectively. The C 1s spectra was normalized to the peak at 284.6 eV by 1.0. (g) The deconvoluted area of the C 1s XPS spectra in (c–f). (h) The ratio of sp^2^ to sp^3^ carbon in GO: sp^2^/(sp^2^ + sp^3^).

The chemical structure of GO powders were investigated by the XPS spectra (Fig. 5). The chemical compositions of GO powders consisted of C, O, and S element (Fig. 5a and Table S3†). Here, the C/O ratio of GO powders recorded much lower values that ranges from 1.38 to 2.05 (Fig. 5b) than the previous literature resulted in the range of 2.0–2.7 as summarized in Table S3.† Thus, the synthesized GO powders obtained higher degree of oxidation. First the degree of oxidation was compared with respect to the graphite sources that have never been dealt with before. The average value was lower in the SPG-based GO powders, which means that the using SPG reached higher degree of oxidation than NFG as well as recorded lower position comparable to the general range (1.5 to 2.5).^24–29^ Even GO powders from SA325P and AA325P apparently show no differences (Fig. S1a and b†), the C/O ratio of SA325P-HGO+ and -IGO were lower than AA325P-HGO+ and IGO; using graphite with higher carbon purity is favorable to reach higher degree of oxidation. Also, it was observed that IGO exhibited the highest degree of oxidation; HGO+, and HGO followed in consistence with the data in previous literature in which GO-TO exhibited the lowest C/O ratio (1.95) between the GOs from the other oxidation methods.^29^Fig. 5c–f display the C 1s XPS spectra of GO powders normalized to the peak at 284.6 eV by 1.0. The C 1s XPS spectra were deconvoluted to calculate the amount of each chemical bond including sp^2^ (284 eV), sp^3^ (284.6 eV), epoxy/hydroxyls (C–O, 286.6 eV), carbonyl (C=O, 288 eV), and carboxylates (O–C=O, 289.2 eV) bonds; the calculated areas of peaks are summarized Fig. 5g and Table S4.† The double amount of oxidant resulted that the epoxy/hydroxyl peak be more intensive in HGO+ and IGO. The GO powders from HGO+ show the lowest carbon amount (sp^2^ + sp^3^) under 36% (Fig. 5g) and the IGO shows slightly higher amount due to use of H_3_PO_4_ as second acid. GO powders from SA100F present the lower oxygen functionalities, compared to the other due to large size of graphite source that hinder the exposure of carbon atoms. AA325F exhibited the higher degree of oxidation due to the shorter diffusion route along the smaller lateral size.^28^ In the case of SPG based GOs, accordingly, they exhibited the highest degree of oxidation because the microscale gaps exited on the surface of SPG that synthesized from amorphous carbon.^30^ Because of broaden shoulder of peak at 284.6 eV, the peak was deconvoluted by sp^2^ and sp^3^ carbon, and their ratio (sp^2^/(sp^2^ + sp^3^)) was compared in Fig. 5h. It was observed that the ratio of sp^2^ increases in the order of HGO, and HGO+, and IGO improves it more; that means the basal sp^2^ hybridized chemical structure of GO remains without transforming into sp^3^.

**Fig. 6:**
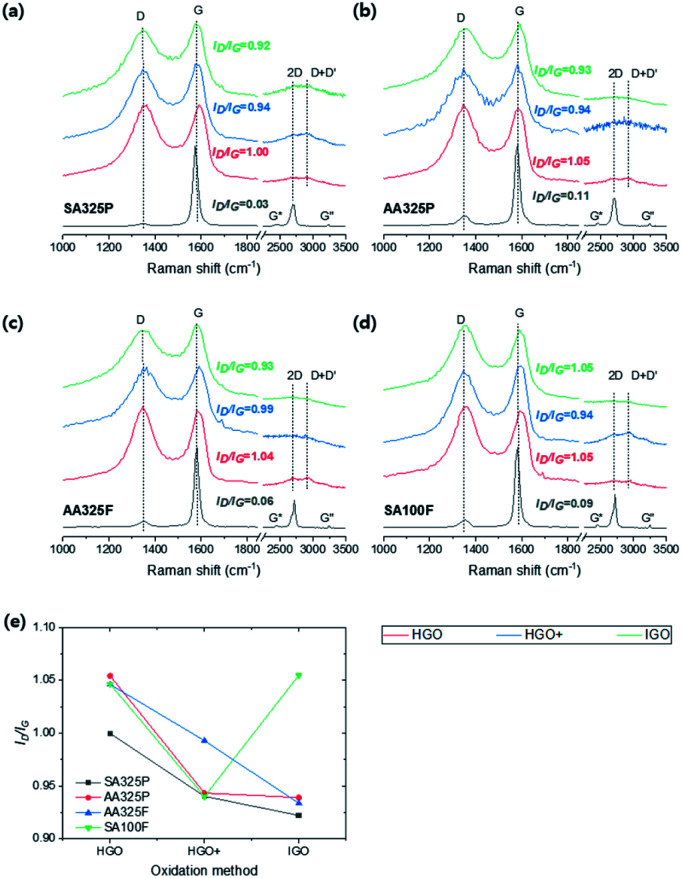
(a–d) Raman spectra of graphite and GOs from SA325P, AA325P, AA325F, and SA100F, respectively. (e) The intensity ratio of D to G band (*I*_D_/*I*_G_) of GOs.

The Raman spectra (*E*_laser_ = 532 nm) of graphite and GOs were measured (Fig. 6). As shown in Fig. 6a–d, all of the spectra exhibit D and G band at ∼1340 and 1580 cm^−1^ due to disorder and the doubly degenerate zone-center phonon E_2g_ mode by C–C stretching,^31^ and 2D region consisted of G*, 2D, D + D′, and G′′. In the spectra of graphite sources, SA325P present the lowest D band; the intensity ratio of D and G band (*I*_D_/*I*_G_) was 0.03, 0.11, 0.06, and 0.09 for SA325P, AA325P, AA325F, and SA100F, respectively. In the 2D region, SPGs exhibit a broader peak at 2D than the peak of NFGs. Fig. 6e shows the trends of *I*_D_/*I*_G_ of GO samples with different oxidation methods. The *I*_D_/*I*_G_ of GO samples decrease in the order of HGO, HGO+, and IGO except for the GO from SA100F. The basal defect of GO can be triggered by many reasons such as oxidative cleavage of a C–C double bond *via* manganese cyclic ester, oxidative cleavage of a ketone (C=O) forming one carboxylic acid (C(=O)OH) and one ketone and acid-catalyzed hydrolysis of an epoxy (C–O–C) producing two hydroxyl bonds.^19^ Here, the addition of H_3_PO_4_ as second acid can prevent the oxidative cleavage by protecting the vicinal diols, and eventually, it minimized defect formation.^32^ The higher sp^2^ ratio of IGO from the XPS C 1s spectra (Fig. 5h) supports this idea: the less transformation into sp^3^ hybridizations. However, the *I*_D_/*I*_G_ of SA100F-IGO is higher than SA100F-HGO+. We think that the deteriorated defect in SA100F-IGO infers that the effect of second acid is weakened because the H_3_PO_4_ with larger molecular size cannot be effectively intercalated into the surface of less exposed large-size NFG.

### 3.3. Thermal expansion of GO

**Fig. 7:**
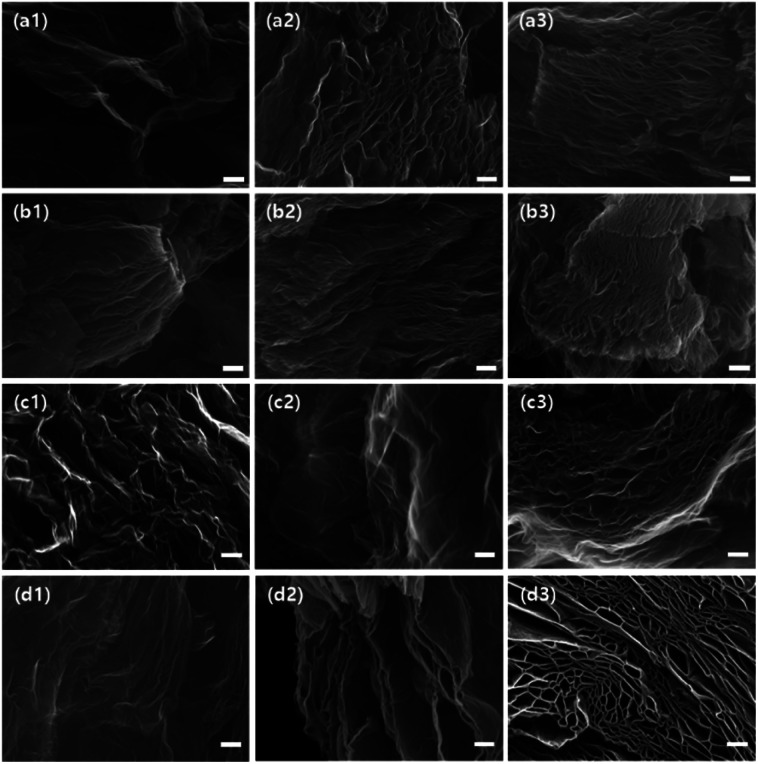
High resolution SEM image (×50 000) of TEGOs. The numbers indicate oxidation methods: 1–3 by HGO, HGO+, and IGO, respectively. Graphite sources from (a1–3) SA325P; (b1–3) AA325P; (c1–3) AA325F; (d1–3) SA100F. The scale bar is 200 nm.

To build GO assembly, the thermal expansion as a top-down approach was applied to the GO powders and converted them to TEGOs. The microstructure of TEGO was characterized by high resolution SEM image (Fig. 9). Because of gas release by thermal reduction of oxygen functional groups, the interlayer of GO was exfoliated and eventually formed a multilayered open porous structure that has the pores with several hundreds of nanometers. The apparent porous structures became denser according to GO powders from HGO to IGO (Fig. 7a(1–3)). In the case of SA325P, AA325P, and AA325F, this trend was similarly observed regardless of graphite source. In the case of the TEGO from SA100F-HGO (Fig. 7d(1) and S3a†), the graphitic layers were exfoliated, and the wrinkled and porous structures are only found at the edge. This might be because the lower oxidation was not enough to trigger the interlayer expansion at the center of the primary particle. The higher oxidation in SA100F-HGO+ and -IGO made the interlayer expansion possible (Fig. S3b and c†).

**Fig. 8:**
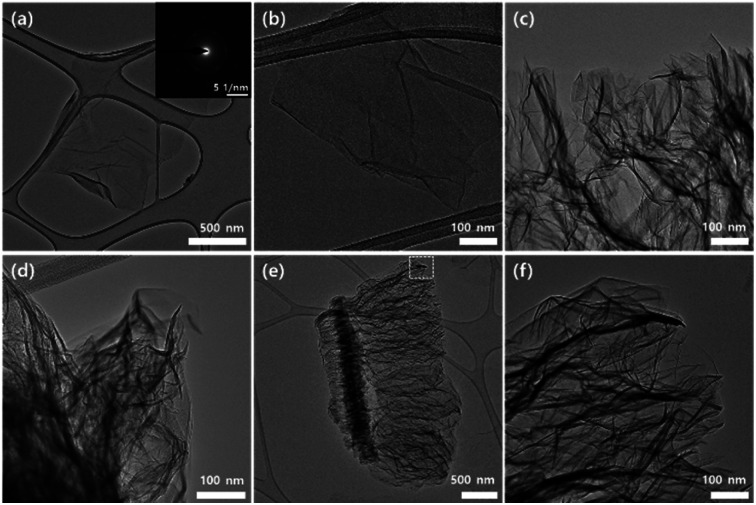
TEM image of TEGOs. A lacey carbon grid was used (a) GO. The inset image is the SAED pattern of GO. TEGO treated by annealing temperature at (b) 100 °C; (c) 200 °C; (d) 400 °C; (e and f) low and high magnification TEM image of TEGO at 600 °C.

To check the interlayer expansion, the microstructure of TEGO was characterized by TEM and SEM according to the annealing temperature (Fig. 8 and S4†). All samples were prepared by immersing TEM grid into GO and TEGO dispersions in ethanol treated by mild sonication. First, the hexagonal array of selected area electron diffraction (SAED) pattern reveals each exfoliated layer is GO sheet (inset of Fig. 8a). The GO morphology remains under temperature at 100 °C (Fig. 8b); and the wrinkled sheets are found above 200 °C (Fig. 8c–f) that indicates thermal expansion triggering temperature. In the case of TEGO from 600 °C, we can observe the nice expanded structure following normal direction of layer orientation.

**Fig. 9:**
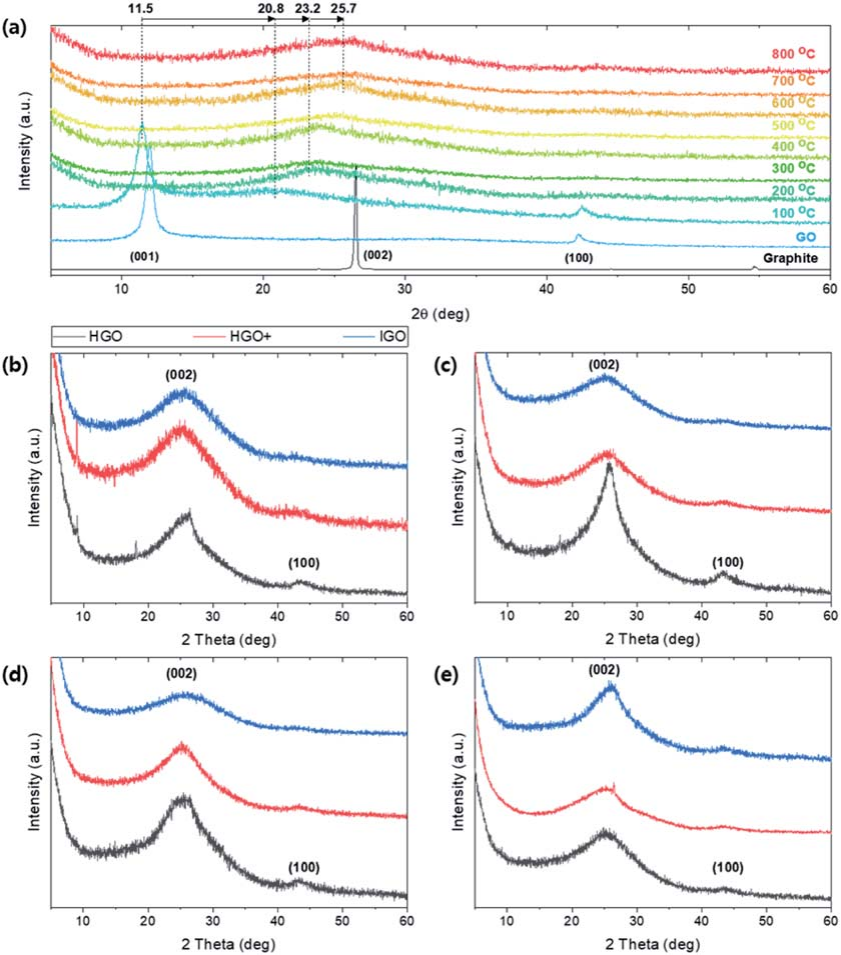
(a) XRD patterns of TEGO annealed at various temperature. (b–e) XRD patterns of TEGOs converted from different graphite sources and oxidation methods: SA325P, AA325P, AA325F, and SA100F, respectively.

Crystalline structural change of TEGO under thermal reduction was monitored by XRD pattern (Fig. 9). The (001) peak at 11.5 degree of GO gradually shifted to (002) at 25.7 degree as the annealing temperature increases (Fig. 9a). At 100 °C, the peak at (001) and partially reduced (002) peak co-exist because the oxygen functional group in the interlayer of graphene starts to be reduced and escape from the spacing. Broad peak is observed at 25–26 degrees above 600 °C. The broaden and shifted XRD patterns are observed regardless of graphite sources (Fig. 9b–e). In the case of TEGO from HGO, (002) peaks are sharper than the TEGO from HGO+ and IGO; the peak near 43.5 degrees corresponding to (100) lattice remains clearly and faded in HGO+ and IGO. Thus, it can be deduced that the reduced and well-exfoliated structure of TEGO from HGO+ and IGO induced the crystal structure more amorphous.

**Fig. 10:**
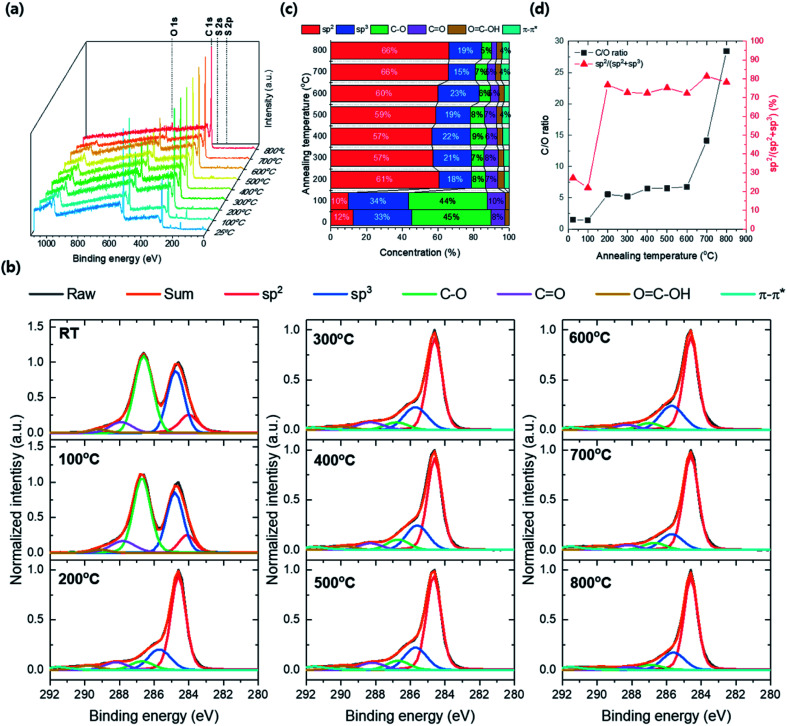
XPS data from GO to TEGO conversion by various annealing temperatures. (a) XPS survey spectra. (b) C 1s XPS spectra. (c) Chemical compositions calculated from the area of deconvoluted peaks. (d) C/O ratio and sp^2^ carbon ratio.

Fig. 10a shows the XPS survey spectra by annealing temperature from 25 to 800 °C. The atomic peaks corresponding to C 1s, O 1s S 2s, and S 2p are confirmed and the peak's intensity changes by annealing temperature. The intensities of S 2s and S 2p are no more observed and the intensity of O 1s dramatically decreases as annealing temperature above 200 °C. Thus, the contaminant S composition was removed and the GO's reduction was induced at the temperature range between 100 and 200 °C. The C 1s XPS spectra for annealing temperature were deconvoluted by carbon bonding structure with sp^2^, sp^3^, C–O, C=O, O=C–OH and π–π* satellites stacking, respectively, and the concentrations of carbon bonding structures were converted from the area occupied by each deconvolution (Fig. 10b and c). The intensity of the C–O peak significantly decreased at 200 °C and the total carbon composition increases over 79% (61 and 18% of sp^2^ and sp^3^, respectively). Besides, the sp^2^ composition in TEGO reaches over 57% after the annealing temperature up 200 °C. The C/O ratio was converted from the atomic concentration, and the sp^2^ carbon ratio, sp^2^/(sp^2^ + sp^3^) was calculated with respect to annealing temperature (Fig. 10d). The C/O ratio jump is found at the 200 °C and maintained until 600 °C, and it drastically increases after 700 °C. The sp^2^/(sp^2^ + sp^3^) fraction highly jumps from 7 to 77% at 200 °C and saturates approximately 75% on average until 800 °C.

### 3.4. Porous characteristic of TEGO

The porous structure of TEGOs were quantitatively characterized by BET analysis.

**Fig. 11:**
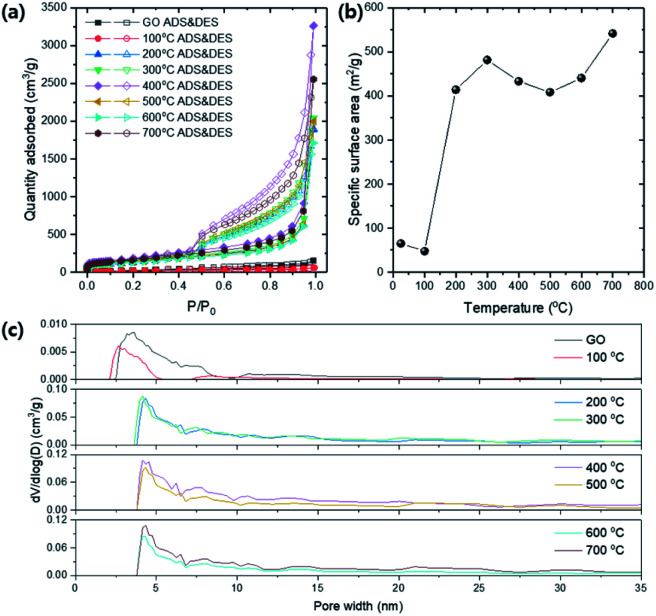
The porous characteristics of TEGOs by various annealing temperatures. (a) N_2_ isotherms at 77 K (ADS: adsorption and DES: desorption). (b) Specific surface area (SSA) *vs.* annealing temperature. (c) Pore size distributions (PSDs) of TEGOs *via* density functional theory (DFT) method.

Fig. 11a shows N_2_ isotherm of GO and TEGO at 77 K regarding various annealing temperatures. Typical mesoporous isotherm curves are obtained, which have hysteresis for the relative pressures from 0.45 to 1.0. The BET specific surface area (SSA) was calculated for the relative pressures in the range from 0.05 to 0.30 (Fig. 11b). The BET SSA of the TEGOs is dramatically increased at 200 °C due to thermal reduction which corresponds to the result confirmed from the XRD and XPS spectra. Many pieces of literature reported that the expansion in the interlayer of graphene is triggered by gas releases during thermal reduction, including CO, CO_2_, and vapors from the moistures trapped in the GO powder.^33,34^ The BET SSA of the TEGO ranges 414–481 m^2^ g^−1^ up to 600 °C, and a slight increase is found at 700 °C. The pore size distributions (PSD) of the TEGOs are calculated by the density functional theory (DFT) method and compared concerning the annealing temperature (Fig. 11c). The pore width of GO is distributed from 2 to 10 nm which can be classified as mesopore. After thermal expansion at 200 °C, the peak position in the PSD is shifted to near 4.5 nm and broadly distributed until 35 nm.

**Fig. 12:**
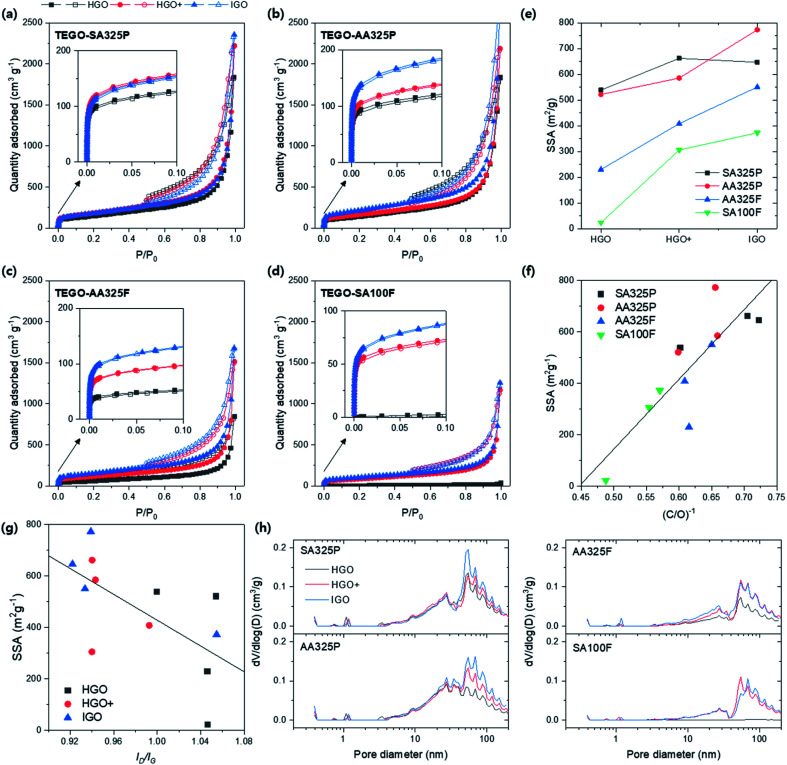
BET analysis of TEGOs converted from different graphite sources and oxidation methods with fixed annealing temperature at 600 °C. (a–d) N_2_ isotherms at 77 K with respect to graphite sources: SA325P, AA325P, AA325F, and SA100F, respectively. Solid symbol: adsorption; empty symbol: desorption. (e) BET SSA of TEGOs. (f) SSA *vs.* the inverse of C/O ratio of corresponding GO, (C/O)^−1^, indicating degree of oxidation. (g) SSA *vs. I*_D_/*I*_G_ of GO. (h) PSDs of TEGOs *via* DFT method.

As a final step, all of the porous structure of TEGOs obtained at 600 °C were quantitatively identified by N_2_ isotherm at 77 K (Fig. 12a–d). All isotherm curves present IV type mesoporous characteristic, but have different quantity adsorbed. The isotherms show two of aspects: the overall adsorbed quantity was highly reached in TEGOs converted from SPGs (Fig. 12a and b) than NFGs; the monolayer saturations were more effective in the order of TEGOs converted from HGO, HGO+, and IGO (inset of Fig. 12a–d). This observation corresponds to the TEGO that was previously converted from GO *via* Tour's method on the amorphous graphite,^35^ and eventually exhibited the higher SSA up to 437.6 m^2^ g^−1^ (Table S5†). The calculated BET SSAs of TEGOs were displayed in Fig. 12e. The SPG-based TEGOs (SA325P and AA325P) exhibit higher SSAs that distribute from 500 to 773 m^2^ g^−1^; the AA325F based TEGOs have lower BET SSA (230–550 m^2^ g^−1^) and the SA100F (23–373 m^2^ g^−1^) follows in order. The maximum SSA of TEGO was over 1.7 times higher than the other references reported. When we consider TEGO under different oxidation methods with same graphite source,^36–38^ it was observed that the BET SSAs increase in the order of HGO, HGO+, and IGO. If we remind the XPS spectra of GOs (Fig. 10), the reduction and release of the intercalated oxygen functional groups trigger the thermal expansion of the graphitic multilayers. Fig. 12f displays the relationship between inverse of C/O ratio of GOs and the SSA of TEGOs with linear fit. Herein, it can be noted that reaching higher degree of oxidation during GO synthesis should be firstly satisfied to improve SSA of TEGO. Considering defect on GO, Fig. 12g displays the relationship between *I*_D_/*I*_G_ of GOs and the SSA of TEGOs. The TEGO converted from the GO that had lower *I*_D_/*I*_G_ recorded higher SSA; IGO was the best strategy, and HGO+ and HGO followed. Among those IGOs, SA100F-IGO did not have consistency with the others due to higher *I*_D_/*I*_G_. To build TEGO with high SSA, hence, the GO should be prepared with proper oxidation that aims higher oxidation and improved crystalline disorder in GO. Finally, the DFT-PSDs of the TEGO powders are compared in Fig. 12h. The peak positions are commonly observed at 50 nm, and broad distribution can be found from 50 to 200 nm that can be identified as primary porous structure in TEGO (Fig. 7); the TEGO has meso- and macroporous characteristics. The PSD over 30 nm was sensitively changed with respect to the oxidation methods. Among the SPG-based TEGOs, the PSD near 30 nm increased as the AA325P was used. NFG-based TEGOs present less PSD under 20 nm, and the PSD of the TEGO from SA100F-HGO was difficult to be confirmed due to low SSA.

## 4. Conclusions

In conclusion, GO synthesis was conducted under three oxidation methods, *i.e.* HGO, HGO+, and IGO by using various graphite sources that based on SPGs and NFGs with different size and supplier. The morphology, crystal structure, and chemical composition of GOs were studied by SEM, TEM, XRD pattern, Raman spectra, and XPS spectra. Degree of oxidation of GO was significantly dependent on the oxidant intercalation behaviour through the unique structure of graphite source; the use of SPGs and smaller particle size was more favourable to attain higher degree of oxidation (*e.g.* C/O ratio < 1.4). Also, the oxidation method should be carefully applied to obtain higher degree of oxidation as well as improved quality in crystalline structure that can be identified by sp^2^ carbon ratio and *I*_D_/*I*_G_ from XPS, and Raman spectra, respectively. In the case of IGO, the addition of H_3_PO_4_ as second acid was effectively exhibit not only higher degree of oxidation but also reduced crystalline disorder because it functioned a protection that prevent –OH cleavage from the basal plane of graphene, resulting higher sp^2^ carbon ratio (>50%). As a top-down approach, the TEGO, porous graphene assembly was obtained by annealing the GO powders to thermally expand graphitic interlayers. The thermal expansion triggered at 200 °C; and the porous microstructure was confirmed by high resolution SEM and TEM. XRD pattern and XPS spectra revealed the TEGO was successfully converted from GO due to thermal reduction. All GO powders were converted to TEGOs at 600 °C, and their porous structure was quantitatively characterized by BET analysis. The degree of oxidation, inverse of C/O ratio, and SSA were linearly fitted. Besides, the lower *I*_D_/*I*_G_ was beneficial to achieve higher SSA up to 773 m^2^ g^−1^. Thus, the choice of starting graphite source and oxidation method can significantly affect to modify structural feature of final product; they should be considered as a major factor to build a graphene assembly from GO.

## Conflicts of interest

The authors declare no competing financial interest.

## Supplementary Material

RA-011-D0RA10207E-s001
