# Supplementary material for: Building with graphene oxide: effect of graphite nature and oxidation methods on the graphene assembly
Source: RSC Adv. 2021 Jan 18;11(6):3645–54. doi: 10.1039/d0ra10207e (PMC8694235; doi:10.1039/d0ra10207e)
Supplement: RA-011-D0RA10207E-s001 [file RA-011-D0RA10207E-s001.pdf]

Electronic Supplementary Material (ESI) for RSC Advances.
This journal is © The Royal Society of Chemistry 2021

Electronic Supplementary Information (ESI)

Building with graphene oxide: effect of graphite nature and oxidation methods on the graphene assembly

Ji Hoon Kim,[a] Gyu Hyeon Shim,[a] Thi To Nguyen Vo,[a] Boyeon Kweon,[a] Koung Moon Kim[a] and Ho Seon Ahn,*[a]

[a]Department of Mechanical Engineering, Incheon National University, Incheon 22012, Republic of Korea.

***Please address all correspondence to**

Associate Professor, Ho Seon Ahn
Department of Mechanical Engineering
Incheon National University
Incheon, 22012, Republic of Korea
E-mail : hsahn@inu.ac.kr

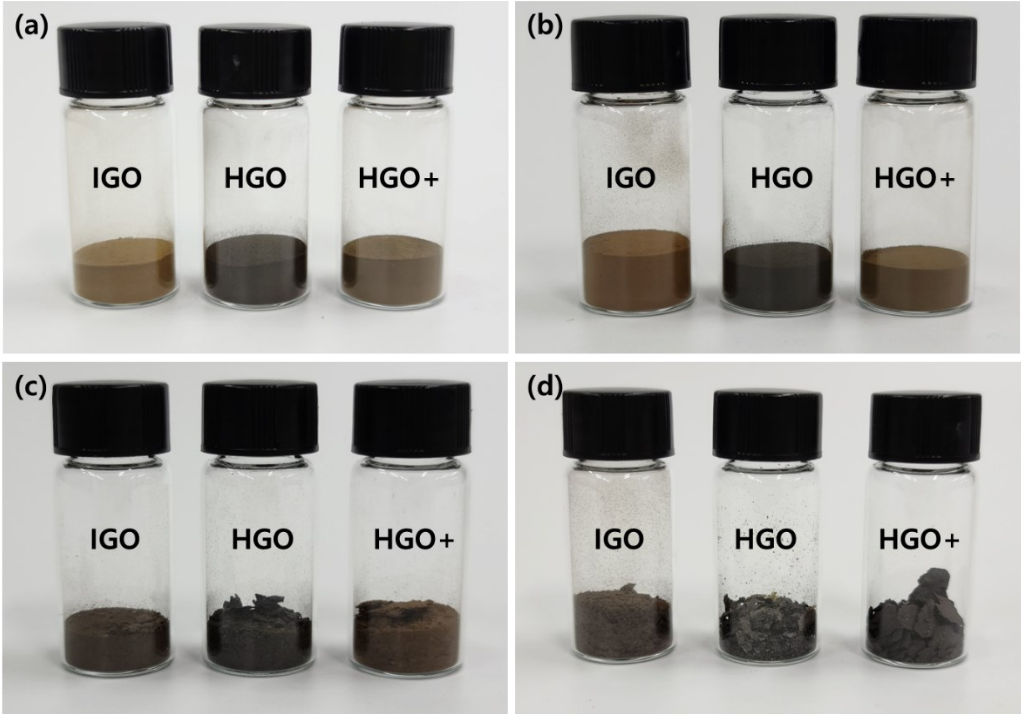

Fig. S1. Digital images of GO powders. Three different oxidation methods (IGO, HGO, and HGO+, respectively) are applied to graphite sources; (a) SA325P, (b) AA325P, (c) AA325F, and (d) SA100F.

Table S1. Digital images of GO powders. Three different oxidation methods (IGO, HGO, and HGO+, respectively) are applied to graphite sources; (a) SA325P, (b) AA325P, (c) AA325F, and (d) SA100F.

Case	Graphite supplier	Graphite size (mesh, size; um)	Graphite type	Oxidation
SA325P-HGO	Sigma Aldrich (SA)	325 (45 um)	Synthetic, powder	HGO
SA325P-HGO+	Code: 496596			HGO+
SA325P-IGO	≥99.99%			IGO
AA325P-HGO	Alfa aesar (AA)	325 (45 um)	Synthetic, powder	HGO
AA325P-HGO+	Code: 10129			HGO+
AA325P-IGO	99%			IGO
AA325F-HGO	Alfa aesar (AA)	325 (45 um)	Natural, Flake	HGO
AA325F-HGO+	Code: 43209			HGO+
AA325F-IGO	99.8%			IGO
SA100F-HGO	Sigma Aldrich (SA)	100 (150 um)	Natural, Flake	HGO
SA100F-HGO+	Code: 808091			HGO+
SA100F-IGO	99%			IGO

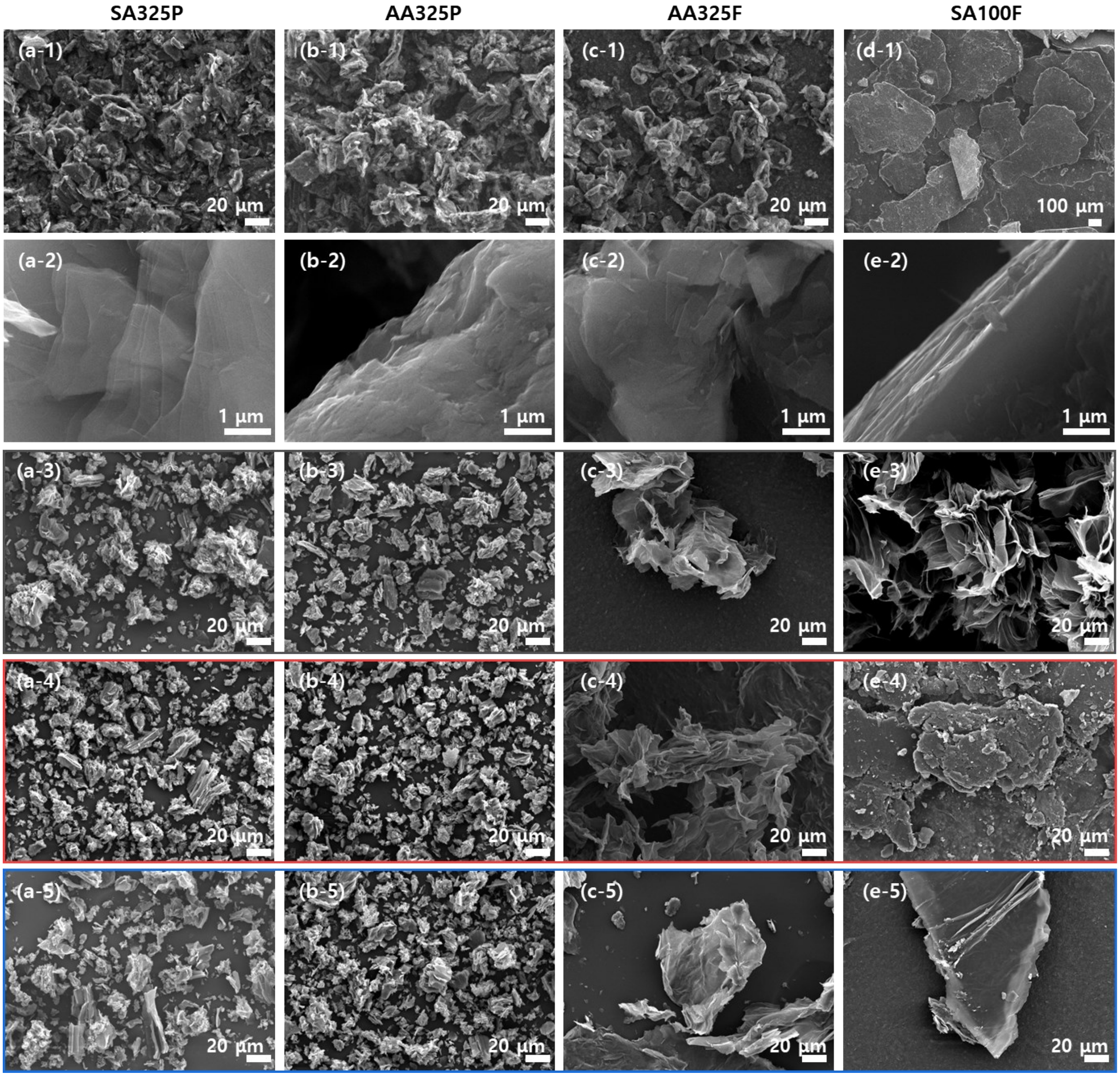

Fig. S2. SEM image of graphite source (a-d) and GO by different oxidation method. (1-2) Graphite with different magnification of x500 and x20,000. (3-5) Low magnification SEM image (x500) of GO from HGO, HGO+, and IGO, respectively.

Table S2. Summaries of d spacing calculated by applying Bragg's law from the XRD patterns (wavelength = 1.5412 Å)

Material	Name	Crystallite (Å)			
		d_{001}	d_{002}	d_{101}	d_{200}
Graphite	SA325P	-	3.375	2.135	2.039
	SA100F	-	3.387	2.089	2.019
	AA325P	-	3.375	2.133	2.047
	AA325F	-	3.362	2.135	2.036
GO	SA325P HGO	7.968	-	2.141	-
	SA325P HGO+	8.114	-	2.141	-
	SA325P IGO	7.968	-	2.141	-
	AA325P HGO	8.188	-	2.141	-
	AA325P HGO+	8.422	-	2.141	-
	AA325P IGO	7.561	-	2.141	-
	AA325F HGO	7.897	-	2.141	-
	AA325F HGO+	7.759	-	2.141	-
	AA325F IGO	8.040	-	2.141	-
	SA100F HGO	7.692	-	2.131	-
	SA100F HGO+	8.754	-	2.140	-
	SA100F IGO	7.625	-	2.121	-

Table S3. Chemical composition of graphite oxide via XPS atomic concentration (at%), and comparision of C/O ratio with other references.

Graphite	Oxidation	Graphite size (μm)	C	O	S	C/O ratio	Ref.
SA325P	HGO	45	61.56	37.06	1.38	1.66	This work
	HGO+	45	57.69	40.67	1.63	1.42	This work
	IGO	45	56.78	41.02	2.20	1.38	This work
AA325P	HGO	45	61.70	36.95	1.35	1.67	This work
	HGO+	45	59.52	39.23	1.25	1.52	This work
	IGO	45	59.88	39.27	0.85	1.52	This work
AA325F	HGO	45	60.94	37.50	1.57	1.63	This work
	HGO+	45	61.65	37.53	0.82	1.64	This work
	IGO	45	59.88	38.93	1.19	1.54	This work
SA100F	HGO	150	66.53	32.46	1.01	2.05	This work
	HGO+	150	63.62	35.27	1.12	1.80	This work
	IGO	150	62.21	35.5	2.29	1.75	This work
SP-1	Hummers	30	-	-	-	2.7	[1]
-	Hummers (HGTO)	30	70	30	-	2.33	[2]
-	Hummers (MGTO)	30	69.47	30.53	-	2.28	
-	Hummers (MGTO3)	30	69.27	30.73	-	2.25	
Expanded graphite	Hummers (EGO)	5	-	-	-	1.39	[3]
Flake graphite	Hummers (FGO)	5	-	-	-	2.03	
Mycroscrystalline graphite	Hummers (MGO)	5	-	-	-	2.07	
NFG-100	Hummers	150	-	-	-	2.70	[4]
NFG-325	Hummers	45	-	-	-	2.35	

NFG-2000	Hummers	6.5	-	-	-	1.67	
-	Staudenmaier (GO-ST)	20	-	-	-	2.47	[5]
-	Hofmann (GO-HO)	20	-	-	-	2.71	
-	Modified Hummers (GO-HU)	20	-	-	-	2.05	
-	Tour (GO-TO)	20	-	-	-	1.95	

Table S4. The relative amount of carbon chemical bonds calculated from the deconvoluted C1s XPS spectra.

Graphite	Oxidation	sp^2 (%)	sp^3 (%)	C-O (%)	C=O (%)	O-C=O (%)	$sp^2/(sp^2+sp^3)$ (%)
SA325	HGO	14.30	25.78	48.48	8.90	2.54	35.67
	HGO+	13.90	17.68	57.94	7.46	3.02	44.01
	IGO	19.47	19.24	50.54	6.51	4.25	50.30
AA325P	HGO	13.54	23.80	52.32	8.52	1.83	36.26
	HGO+	15.46	18.50	56.54	7.75	1.75	45.52
	IGO	18.80	16.92	51.81	9.80	2.67	52.62
AA325F	HGO	14.62	30.76	44.54	7.84	2.23	32.22
	HGO+	14.04	18.79	53.28	10.53	3.36	42.76
	IGO	19.30	16.94	52.66	8.17	2.93	53.25
SA100F	HGO	15.64	32.40	42.05	7.50	2.41	32.56
	HGO+	15.41	24.44	49.30	7.68	3.16	38.67
	IGO	18.02	27.77	45.67	6.47	2.08	39.35

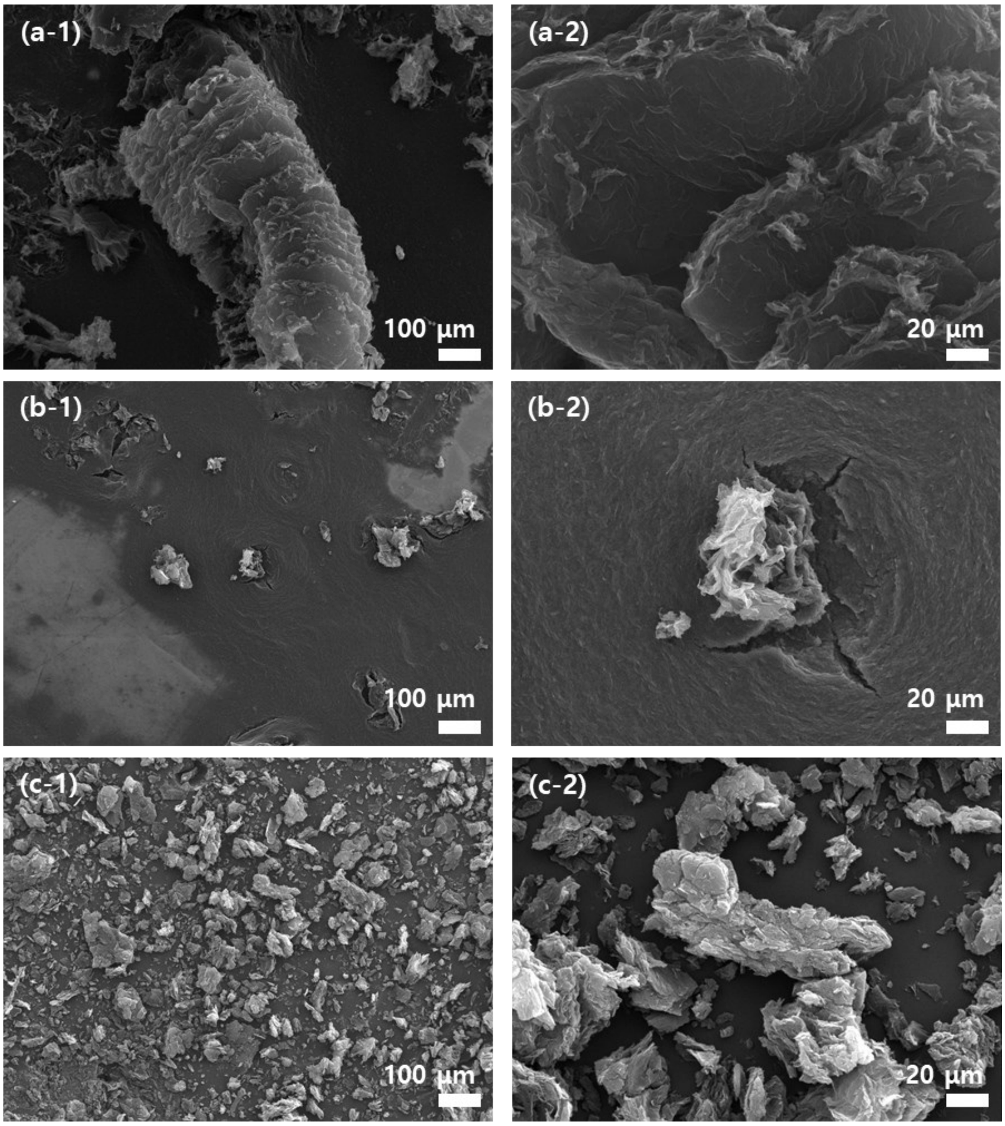

Fig. S3. SEM image of thermally expanded graphene oxide (TEGO) from SA100F with (a) HGO, (b) HGO+, and (c) IGO with different magnifications: (1-2) x500 and x10,000, respectively.

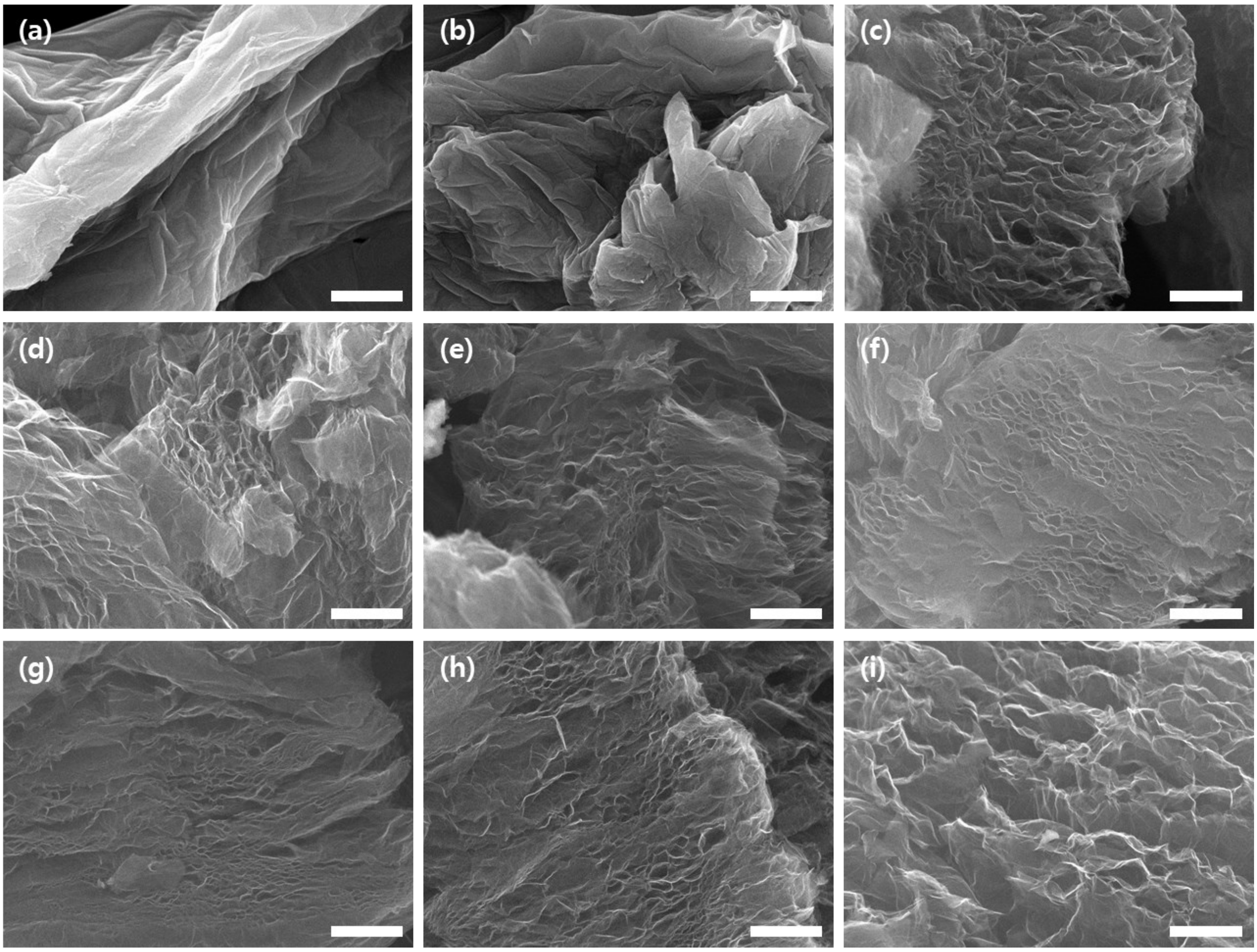

Fig. S4. High magnification SEM image of (a) GO, and (b-i) TEGO powders by annealing temperature 100, 200, 300, 400, 500, 600, and 700 ℃, respectively.

Table S5. Comparison of the SSA of TEGO by the starting graphite source, oxidation method, and C/O ratio of GO with the other references.

Starting graphite	Oxidation method	C/O ratio of GO	SSA (m^2/g)	Ref.
Graphite (450 nm)	Hummers	-	62.2 – 403.7	[6]
-	Hummers	1.8	300	[7]
Amorphous graphite	Tour	-	75 – 437.62	[8]
Graphite powder (5 – 20 μm)	Hummers	2.01	46 - 248	[9]
SPG, NFG (<45 & 150 μm)	HGO<br>HGO+<br>IGO	1.38 – 2.05	500 - 773	This work

References

[1] S. Stankovich, D.A. Dikin, R.D. Piner, K.A. Kohlhaas, A. Kleinhammes, Y. Jia, Y. Wu, S.T. Nguyen, R.S. Ruoff, Synthesis of graphene-based nanosheets via chemical reduction of exfoliated graphite oxide, carbon 45(7) (2007) 1558-1565.

[2] G. Shao, Y. Lu, F. Wu, C. Yang, F. Zeng, Q. Wu, Graphene oxide: the mechanisms of oxidation and exfoliation, Journal of materials science 47(10) (2012) 4400-4409.

[3] X. Hu, Y. Yu, J. Zhou, L. Song, Effect of graphite precursor on oxidation degree, hydrophilicity and microstructure of graphene oxide, Nano 9(03) (2014) 1450037.

[4] L. Shen, L. Zhang, K. Wang, L. Miao, Q. Lan, K. Jiang, H. Lu, M. Li, Y. Li, B. Shen, Analysis of oxidation degree of graphite oxide and chemical structure of corresponding reduced graphite oxide by selecting different-sized original graphite, RSC advances 8(31) (2018) 17209-17217.

[5] C.K. Chua, Z. Sofer, M. Pumera, Graphite oxides: effects of permanganate and chlorate oxidants on the oxygen composition, Chemistry–A European Journal 18(42) (2012) 13453-13459.

[6] J.M. Kim, W.G. Hong, S.M. Lee, S.J. Chang, Y. Jun, B.H. Kim, H.J. Kim, Energy storage of thermally reduced graphene oxide, International journal of hydrogen energy 39(8) (2014) 3799-3804.

[7] Y. Qiu, F. Guo, R. Hurt, I. Külaots, Explosive thermal reduction of graphene oxide-based materials: mechanism and safety implications, Carbon 72 (2014) 215-223.

[8] I. Sengupta, S. Chakraborty, M. Talukdar, S.K. Pal, S. Chakraborty, Thermal reduction of graphene oxide: How temperature influences purity, Journal of Materials Research 33(23) (2018) 4113-4122.

[9] S.B. Singh, M. De, Thermally exfoliated graphene oxide for hydrogen storage, Materials Chemistry and Physics 239 (2020) 122102.
